# Prostate Cancer Severity Associations with Neighborhood Deprivation

**DOI:** 10.1155/2011/846263

**Published:** 2011-10-09

**Authors:** Charnita M. Zeigler-Johnson, Ann Tierney, Timothy R. Rebbeck, Andrew Rundle

**Affiliations:** ^1^Center for Clinical Epidemiology and Biostatistics, University of Pennsylvania Perelman School of Medicine, Philadelphia, PA 19104, USA; ^2^Abramson Cancer Center, University of Pennsylvania, Philadelphia, PA 19104, USA; ^3^Mailman School of Public Health, Columbia University, New York, NY 10027, USA

## Abstract

*Background*. The goal of this paper was to examine neighborhood deprivation and prostate cancer severity. *Methods*. We studied African American and Caucasian prostate cancer cases from the Pennsylvania State Cancer Registry. Census tract-level variables and deprivation scores were examined in relation to diagnosis stage, grade, and tumor aggressiveness. *Results*. We observed associations of low SES with high Gleason score among African Americans residing in neighborhoods with low educational attainment (OR = 1.34, 95% CI = 1.13–1.60), high poverty (OR = 1.39, 95% CI = 1.15–1.67), low car ownership (OR = 1.46, 95% CI = 1.20–1.78), and higher percentage of residents on public assistance (OR = 1.32, 95% = 1.08–1.62). The highest quartile of neighborhood deprivation was also associated with high Gleason score. For both Caucasians and African Americans, the highest quartile of neighborhood deprivation was associated with high Gleason score at diagnosis (OR = 1.34, 95% CI = 1.19–1.52; OR = 1.71, 95% CI = 1.21–2.40, resp.). *Conclusion*. Using a neighborhood deprivation index, we observed associations between high-grade prostate cancer and neighborhood deprivation in Caucasians and African-Americans.

## 1. Introduction

Prostate cancer is the most prevalent malignant cancer among men in the U.S. 217,730 incident cases were expected in 2010 [1]. The advent of prostatic specific antigen (PSA) testing has driven large increases in diagnoses with dramatic increases observed between 1988 and 1993, coinciding with the advent of widespread PSA testing [2–4]. African Americans have a significantly higher risk of disease than Caucasian men, tend to be diagnosed with more aggressive disease, and suffer the greatest mortality associated with prostate cancer [5]. In spite of its common occurrence and strong racial disparities, modifiable risk factors for prostate cancer have not been confirmed. These disparities are believed to be a result of interactions among genes, health behaviors, and environmental factors.

Economic, physical, and social characteristics of residential neighborhoods may influence health-related behaviors, screening behaviors and health conditions. Disadvantaged neighborhoods are often correlated with higher levels of environmental pollutants, overcrowding, violence, less social cohesion, and less access to services [6]. Of particular importance for diseases such as prostate cancer in which screening practices have had large effects on incidence, low-income neighborhoods often have fewer medical facilities and these facilities are often stressed due to higher burdens of indigent care. The effects of race-based residential segregation may also have a distinct effect on the spatial accessibility of health care facilities [7]. A recent national study showed that in the most segregated counties, a greater proportion of African American residents was associated with a significantly lower volume of outpatient surgery, fewer ambulatory surgery facilities, fewer general surgeons, and a significantly higher volume of emergency medical visits [8].

Only a few studies have investigated the effects of neighborhood economic and social conditions on prostate cancer incidence and aggressiveness at diagnosis. However the extant data suggest that higher socioeconomic status measured at the individual or neighborhood level predicts a higher risk of prostate cancer diagnosis and a lower risk of late-stage disease at diagnosis. The National Program of Cancer Registries Patterns of Care Study found that higher average neighborhood educational attainment and income measured at the Census Tract level is associated with lower-stage prostate cancer at diagnosis [9]. Recent analyses of SEER-Medicare data show that higher zip code level median household income is protective against advanced stage disease at diagnosis [10].

Although socioeconomic and ethnic differences in prostate cancer outcomes persist, no studies of neighborhood level factors have reported on prostate cancer severity as an outcome stratified by race. Additionally, prior studies have tended to focus on single variable indicators of socioeconomic status, for instance percent poverty, which do not necessarily reflect all of the dimensions of socioeconomic stratification across neighborhoods. The aims of this study were: (1) to determine if census tract level SES factors are differentially associated with indicators of prostate cancer severity by race and (2) to determine whether a more comprehensive measure of neighborhood SES more strongly predicted prostate cancer severity than single variable indicators of economic stratification.

## 2. Materials and Methods

### 2.1. Study Participants

Anonymized data from the Pennsylvania Department of Health was provided on prostate cancer patients diagnosed in the Commonwealth of Pennsylvania from 1995 to 2007. In the present analysis, we focused on a sample who resided in Southeastern Pennsylvania, the primary service area of patients at the University of Pennsylvania and representative of the Philadelphia metropolitan area. The geocoded subset of patients focused on Philadelphia county and the surrounding 4 counties (Bucks, Delaware, Montgomery, and Chester). This sample identifies a targeted region with a defined population base representing a variety of sociodemographic conditions of interest to the present analysis. Residential addresses of prostate cancer patients in the Pennsylvania cancer registry were cleaned by trained research staff and geocoded with Arc GIS. A total of 5,136 African American and 16,672 Caucasian men were geocoded from this Philadelphia 5-county region.

### 2.2. Neighborhood Variables

Census data describing the sociodemographic characteristics of the census tracts for the five counties were downloaded from the Census Bureau web site (http://www.census.gov) from 2000 Census Summary File 3. Downloaded data were census tract characteristics of interest for this study. Variables extracted from this database included household income, adult high school educational attainment, percent poverty, percent of female-headed households with dependent children, percent of households with no car, percent of households on public assistance, percent of unemployed adults, percent vacant housing units, percent of homes with more than 1 occupant per room, home value, percent of non-Hispanic Black residents, percent of males in management positions, percent of females in management positions, percent of males in professional occupations, percent of females in professional occupations, percent of rented units, percent of males not in the labor force, percent of total population 65 years and over, percent of residents who did not move since 1995, and percent of renters or owners paying more than 50% of income for home. 

We also calculated a deprivation index based on one originally developed and tested by Messer et al. on several geographic regions in the U S [11]. The index uses a principal components analysis (PCA) approach. The deprivation index was used to facilitate the comparison of neighborhood deprivation and health across geographic areas. Twenty census variables described and selected by Messer et al. were included in our PCA [11]. They characterized SES and demographic domains associated with health outcomes in the literature. The variables that loaded in the top 20 percentile (explaining the greatest amount of variance) were retained for inclusion in the deprivation index. These 5 variables were (1) percent of households with income <$30,000/year, (2) percent poverty, (3) percent of households on public assistance, (4) percent of female head of household with dependent children, and (5) percent of households with no car. A final PCA was run with the 5 retained variables to determine the weight of each variable's contribution to the deprivation score for each census tract in the study area. The weighted deprivation score standardized by SAS to have a mean of 0 and standard deviation of 1 ranged from −1.07 (low deprivation) to +4.02 (high deprivation). Quartiles of continuous neighborhood deprivation were then created.

### 2.3. Outcome Variables

Our primary outcome variables were indicators of prostate cancer severity that are associated with differences in long-term survival [12]. These variables include tumor stage, with low stage defined as stages 1 and 2 (localized disease), and high-stage is defined as stages 3 and 4 (nonlocalized); tumor grade, with low grade is defined as tumor Gleason score of 6 or below and high-grade is defined as a tumor score of 7 or greater; and tumor aggressiveness, defined as a combined high tumor stage (stage 3 or 4) and high tumor grade (grade 7+) compared to those with other combinations of these variables.

### 2.4. Statistical Analyses

*t*-tests were used to compare age means for the groups. *χ* ^2^ (frequency) tables were evaluated using Pearson chi-square tests to determine significant differences by race for categorical patient-level and neighborhood-level variables. Generalized estimating models (GEE) using a logit link function, binomial distributions, and robust standard error estimation were used to estimate odds ratios (OR) for associations between neighborhood socioeconomic measures and prostate outcomes accounting for the clustering of multiple patients within census tracts [13]. Two-sided *P*-values <0.05 were considered significant. 

Stratifying the data by race (African American or Caucasian), frequency tables and GEE models were used to determine which neighborhood variables are associated with prostate cancer outcomes. Multicollinearity is an issue when modeling neighborhood variables, so we examined each neighborhood variable in separate models [14]. We also created GEE models to examine the quartiles of the deprivation index in relation to outcome variables. The first quartile, representing lowest neighborhood deprivation, was the reference group. Additional unstratified analyses (adjusting for African American race compared to Caucasian) were conducted to examine whether racial differences are attenuated when census tract-level variables are added to the models. We adjusted for age group <60 or ≥60 and year of diagnosis (modeled as a continuous variable) in all GEE models.

## 3. Results

### 3.1. Sample Characteristics

Table 1 presents demographic characteristics of prostate cancer patients by race. There were significant ethnic differences for all patient-level variables (*P* < 0.001). Compared to Caucasians, African Americans were younger (66 versus 68 years), less likely to be married (57% versus 77%), and more likely to have unfavorable prostate cancer characteristics (high-stage, 15% versus 12%, and high Gleason Score, 28% versus 22%).

### 3.2. Neighborhood SES Characteristics

Table 1 also presents SES characteristics of the patients' residential census tracts. There were significant ethnic differences for all neighborhood-level variables (*P* < 0.001). Compared to Caucasians patients (38-39%), African Americans (86–89%) were more likely to live in low-SES neighborhoods, characterized by below-sample median income and education. The neighborhoods of African American cases were also more likely to have higher than median percentages of poverty, single female head of households, no car ownership, and households on public assistance.

Table 2 presents neighborhood SES indicators in association with prostate cancer severity outcomes. There were no associations of neighborhood SES with aggressive (high-stage and high-grade) tumor in this subset of cases. However, the prevalence of high-stage prostate cancer was lower in Caucasian men living in neighborhoods with high percentage of residents on public assistance (OR = 0.89, 95% CI = 0.80−0.99). No other associations with stage at diagnosis were observed. 

The strongest associations between Gleason score and neighborhood SES were observed for African Americans. African Americans residing in neighborhoods with high poverty (OR = 1.39, 95% CI = 1.15−1.67), low income (OR = 1.26, 95% CI = 1.05−1.51), low educational attainment (OR = 1.34, 95% CI = 1.13−1.60), more households with no car (OR = 1.46, 95% CI = 1.20−1.78), and higher percentage of residents on public assistance (OR = 1.32, 95% CI = 1.08–1.62) had a higher Gleason score at diagnosis. Except for ≥ median percent of households with no car (OR = 1.09, 95% CI = 1.01−1.19), there were no associations of these individual neighborhood SES indicators and Gleason score among Caucasians.

### 3.3. Neighborhood Deprivation

Tumor aggressiveness was associated with the highest level of neighborhood deprivation in Caucasian patients only (OR = 1.27, 95% CI = 1.01−1.59). The overall *P*-value for neighborhood deprivation for this outcome was not significant (*P* = 0.055). For both Caucasians and African Americans, the highest quartile of neighborhood deprivation was associated with high Gleason score at diagnosis (OR = 1.34, 95% CI = 1.19−1.52; OR = 1.71, 95% CI = 1.21−2.40, resp.; Table 2). The overall *P*-value for neighborhood deprivation for both groups was <0.001. Trend tests were significant only for Gleason score for both Caucasian (*P* ≤ 0.001) and African American patients (*P* = 0.002).

### 3.4. Race Effects

By conducting an unstratified analysis, we observed that African American race was significantly associated with tumor aggressiveness (OR = 1.31, *P* < 0.001), high-stage (OR = 1.27, *P* < 0.001), and high Gleason score (OR = 1.37, *P* < 0.001) at diagnosis (Table 3). The association between race and prostate cancer severity was only slightly attenuated or remained unchanged when neighborhood SES variables were included in the model. The addition of census tract variables, including the deprivation index, to the models did not change the significance level of race (*P* = 0.001) except in the model including neighborhood deprivation in association with tumor aggressiveness. In this model, the odds of patients with aggressive disease being African American was 1.20 but still significant (*P* = 0.020). The interaction between race and the neighborhood deprivation index was not statistically significant for any of the outcomes (*P* = 0.170 for aggressiveness, *P* = 0.622 for stage, and *P* = 0.416 for Gleason). Trend tests showed that increasing deprivation was associated with increased odds of high Gleason score in the combined sample (*P* < 0.001). No significant trends were observed for the other two outcomes.

## 4. Discussion

Our first study aim was to examine if neighborhood SES was differentially associated with prostate cancer severity comparing African American and Caucasian prostate cancer patients. We found that there were differences in observed associations for both groups. There were associations with low neighborhood SES and outcomes involving the Gleason score, primarily among African American cases. Most of these neighborhood variables measure similar SES parameters, so observed associations are expected for multiple variables and in the same direction. Although African Americans are at high risk for advanced prostate cancer, it is interesting that this particular outcome and not stage is so consistently associated with low neighborhood SES only in African Americans. This is the first report that the authors are aware of showing this difference by race and suggests that tumor grade in African Americans may be particularly prone to neighborhood influences. 

The Gleason score may be less affected by screening practices than stage at diagnosis, and therefore may be more closely tied to biological mechanisms of prostate cancer progression. Although speculative, these mechanisms may be genetic or tied to other risk factors that are disproportionately prevalent among African Americans. Obesity is one factor that is more common in African Americans and is associated with a biologically more aggressive form of prostate cancer [15]. Obesity varies by SES factors and, therefore, may be even more relevant in the discussion of prostate cancer disparities. As African Americans are much more likely than Caucasians to live in disadvantaged areas [16], the possibility of an interaction between patient-level variables and neighborhood-level SES is possible. We were not able to test this hypothesis with the data available in this dataset. 

Emerging evidence also indicates that inflammation is a probable pathway for prostate cancer progression [17]. Increased environmental stress is one pathway through which many primary neighborhood factors, such as SES, are believed to exert their effects on the body. It is still unclear what the specific ingredients of a stressful environment that could promote inflammation processes might be. However, the health-modulating effects of chronic stress have been identified as potential pathways that increase risk of disease and may be connected to general SES [18]. Psychosocial stress associated with poverty may increase the risk of many illnesses [19]. In anticipation of an impending challenge, stress that may have been acute (adaptive for our bodies) becomes chronic (pathogenic for our bodies). A prolonged stress response ultimately results in suppressed immunity and impairs disease defenses. Stress can affect reproductive hormones and immune responses. Cellular and molecular events that promote cancer growth also are affected by stress, and DNA repair mechanisms may be impaired because of stress and cancer defense mechanisms may be disrupted. Stress may influence the expression of viral oncogenes and the replication of tumorigenic viruses. It may also promote tumor growth by facilitating the development of blood supply to the tumor [19]. 

Differential exposure to stressors may explain a portion of health disparities that we observe by both race and neighborhood SES. Residential neighborhood factors may capture structural and social context that influence overall health and related behavior. Neighborhood deprivation, deterioration, urbanization, poverty, education, segregation, social disorder, and income have been correlated with disease rates and health outcomes [20–28]. 

We also observed a single inverse association of neighborhood public assistance on stage at diagnosis in Caucasian patients. This finding was unexpected, as it is the only significant, protective relationship observed in these analyses. This neighborhood variable has not been studied in the context of prostate cancer staging or screening. Patient-level data suggests that subsets of patients on Medicaid are at increased risk for late prostate cancer diagnosis [29]. Therefore, it is not clear why our Caucasian subset would be at lower risk for advanced disease if they reside in lower SES neighborhoods. 

Income and education are commonly used in the US as measure of patient- and neighborhood-level SES. Both income and educational attainment have been shown to affect risk for cancer diagnosis. A study using the New Jersey Cancer Registry observed clusters of prostate cancer incidence to be associated with geographic areas with higher percentages of foreign-born persons, higher poverty, and lower education [30]. According to SEER data, higher educational attainment has been associated with greater risk of prostate and breast cancers alike. Compared to college-educated men, men with less than a college education were 0.79 as likely to be diagnosed with prostate cancer. Low-income men (family income <$25,000) were also at lower risk for prostate cancer compared to men with a family income of $50,000+ [31]. Prostate screening (and therefore prostate incidence) has been shown to be more common in men with higher education, white collar jobs, access to good healthcare, urban residences, and higher household income [32]. A similar positive association between neighborhood SES and breast cancer screening behavior has been observed, even after adjusting for distance to screening facility, urban-rural status, and type of screening facility [33]. Both zip code community SES and zip code urbanicity are positively associated with breast cancer incidence, even after adjusting for individual education [27]. 

Although, in general, high SES may be associated with prostate cancer incidence/diagnosis, low-SES is associated with more severe disease at diagnosis, suggesting more likely progression and increased risk of cancer-related mortality. Associations between lower neighborhood SES and advanced stage or grade at diagnosis have been observed previously. Lower income has been associated with late-stage prostate cancer diagnosis in the SEER dataset (*P* = 0.002) [31]. Klassen et al. found that subsets of Caucasian men living in high-income areas were at particular low-risk for aggressive prostate tumors [34]. A prostate cancer study in Australia showed that three-year survival was poorer and use of radical prostatectomy was less in men from socioeconomically and geographically disadvantaged backgrounds [35]. Results from the ARIC Study showed that rates of all-cause death, cardiovascular death, and cancer death were greater for men and women living in the lowest income bracket compared to those in the highest [22]. A multilevel study using Florida state data coupled with medical records demonstrated that in addition to individual factors such as Black race, single marital status, current and former smoking status, and older age, advanced prostate cancer was significantly associated with living in census tracts with a low median income and lower percent of residents with a college education [36]. Our study also showed that African American race remained significant even after including neighborhood SES factors in multivariable analysis.

In addition to single variable associations, neighborhood indices representing socioeconomic disadvantage have been associated with various health outcomes [11]. In our study, we found that Caucasians and African Americans in more deprived neighborhoods were more likely to be diagnosed with high-grade prostate cancer. Consistency of these findings with regard to outcomes involving tumor grade may suggest that the deprivation index captures underlying factors of neighborhood SES that together contribute to advanced prostate cancer risk across ethnic groups. Highest levels of neighborhood deprivation were significantly associated with tumor grade in both ethnic groups. To date, few studies have used a deprivation index to examine prostate cancer severity and/or outcomes. One in the UK found that patients from more deprived neighborhoods were more likely than men from less deprived areas to be diagnosed with late stage (stage III or IV) prostate cancer. As in our study, more deprived patients were older. In multivariable analysis, increased deprivation was significantly associated with lower odds of radiation therapy (OR = 0.92, CI = 0.90−0.94) and surgery (OR = 0.90, 95% CI = 0.87−0.94) [37]. A study of the California Cancer Registry used a composite SES score to evaluate treatment outcomes in prostate cancer patients. Men from low-SES areas that were treated by surgery or radiation had increased odds of cancer-specific death. Men from lower SES areas were also half as likely to undergo radical prostatectomy for low-risk disease. Adjusting for race made these findings even more profound. Together, these results may suggest the need for improved screening and treatment in men from low-SES communities [38].

### 4.1. Study Limitations and Strengths

The limitations of our study include the fact that the cut-points between more and less advantaged neighborhoods are arbitrary and dependent upon our sample characteristics. However, using the deprivation index to examine neighborhood SES will make this study more comparable to future studies that use similar methods. In addition, our study investigated only census tract-level SES variables, ignoring other contextual characteristics that vary by family, social networks, workplace, and other levels of socially/physically bounded measures of community/geography. We may also be limited by the “intersection of racial and SES segregation,” in which there are relatively few African Americans in the least deprived areas and few Caucasians in the most deprived areas [11]. However, among study areas in the study by Messer et al., Philadelphia showed the largest range in deprivation scores [11]. Therefore, studying the Greater Philadelphia area may have provided an opportunity to observe the effects of neighborhood deprivation better than we could have in other urban populations. 

Another limitation of this study is that we were unable to determine the length of time at residency and if there are modifying effects that result from duration of exposure [39]. We do not yet know when neighborhood factors are most likely to contribute to cancer outcomes (during childhood or adolescence, during the period before clinical disease onset or after treatment). We also do not know much about the period of time that is required for a particular neighborhood exposure or set of exposures to affect the biology and progression/recurrence of disease in an individual with prostate cancer [40]. Factors like neighborhood SES can be measured at various time points during the lifespan. The relative time frame depends on presumed exposures, causal pathways, and associated etiologic periods [41]. Thus, we have decided to begin our investigation at the point of prostate cancer diagnosis. This allows us to be consistent across all patients. It also provides a sensible timeframe that may be closely linked to the lifestyle and environmental factors that are most likely to influence prostate cancer progression and outcomes. We were unable to evaluate other patient-level variables related to lifestyle and treatment because we were limited by the data collected by the PA Department of Health for these analyses.

A particular strength of this study is the use of a standardized deprivation scoring system. The use of different and multiple definitions of variables used in previous prostate cancer studies made it difficult to assess the evidence for associations systematically. However, the fact that we find similar associations with prostate cancer when multiple definitions of neighborhood SES are used suggests the validity of these findings across studies and populations. Composite variables are also less likely to be significantly influenced by changes in single contributing variables over time. In addition, making conclusions based on one neighborhood SES factor without considering the status of other related contextual variables may lead to inappropriate conclusions [11]. We were also able to determine relationships between neighborhood deprivation and prostate cancer severity by race. Other studies of neighborhood deprivation and prostate cancer severity have not had the diversity to examine patterns of association stratified by race [42] or have only adjusted for ethnicity in multivariable analyses [38]. Evidence of an association between the environment and prostate cancer outcomes can increase our knowledge about risk factors for prostate cancer and stimulate new ideas about prevention strategies. This research also may identify segments of the population that may benefit from targeting interventions. Because prostate cancer is so common in the general population, even if only a small increased risk of disease is associated with it, the potential for decreasing the overall morbidity and mortality attributable to neighborhood deprivation may be significant.

## 5. Conclusions

The goal of this study was to examine the relationship between neighborhood SES or deprivation and prostate cancer severity in a diverse population of patients representing the general population of Southeastern Pennsylvania. We found significant differences in neighborhood SES by race. We also observed differences in prostate cancer severity by neighborhood SES and higher degree of neighborhood deprivation. The associations were strongest and most consistent for African Americans.

The science of studying health disparities and neighborhood characteristics (from appropriate methods and models to proper outcome measures and results interpretation) is still young. Future analyses examining this deprivation index in other ethnic groups and in multilevel models may help to determine the effect of neighborhood SES on prostate cancer outcomes. Understanding which neighborhood-level variables best predict poor health outcomes in different environmental settings may aid all researchers in unraveling the complexities of prostate cancer disparities in America.

## Figures and Tables

**Table 1:** Demographics of southeastern Pennsylvania cancer registry prostate cancer patients (1995–2007).

	Caucasian (*N* = 16672)	African American (*N* = 5136)	*P* value
Patient-level variables			
Age at diagnosis, mean (SD)	67.6 (8.94)	66.0 (9.21)	<.001
Married	12826 (77%)	2931 (57%)	<.001
High stage (III/IV)	2040 (12%)	785 (15%)	<.001
Gleason score (7+)	3697 (22%)	1441 (28%)	<.001
Aggressive tumor	1053 (6%)	423 (8%)	<.001

Neighborhood-level variables			
≥ Median % neighborhood poverty	6381 (38%)	4582 (89%)	<.001
≥ Median % household income <$30,000	6401 (38%)	4482 (87%)	<.001
< Median % high school education	6478 (39%)	4412 (86%)	<.001
≥ Median % female head of household with dependent child(ren)	6307 (38%)	4607 (90%)	<.001
≥ Median % households with no car	6341 (38%)	4595 (89%)	<.001
≥ Median % public assistance	6319 (38%)	4583 (89%)	<.001

**Table 2:** Stratified analysis—associations of neighborhood SES characteristics with indicators of prostate cancer severity (GEE) adjusted for age and diagnosis year.

Effect	Tumor aggressiveness		High stage		High Gleason	
	Caucasian OR (95% CI)	African American OR (95% CI)	Caucasian OR (95% CI)	African American OR (95% CI)	Caucasian OR (95% CI)	African American OR (95% CI)
≥ Median % neighborhood poverty	0.98 (0.86, 1.12)	1.08 (0.79, 1.48)	0.92 (0.83, 1.03)	0.97 (0.78, 1.22)	1.05 (0.97, 1.14)	1.39*** (1.15, 1.67)
≥ Median % household income <$30,000	1.06 (0.93, 1.22)	0.98 (0.74, 1.29)	1.01 (0.91, 1.12)	0.99 (0.80, 1.23)	1.08 (0.99, 1.17)	1.26* (1.05, 1.51)
< Median % high school education	1.12 (0.99, 1.28)	1.14 (0.87, 1.48)	1.01 (0.91, 1.13)	1.02 (0.84, 1.24)	1.07 (0.98, 1.15)	1.34** (1.13, 1.60)
≥ Median % female head of household with dependent child(ren)	1.03 (0.90, 1.18)	0.97 (0.71, 1.32)	0.94 (0.84, 1.04)	1.00 (0.79, 1.27)	1.07 (0.99, 1.16)	1.18 (0.97, 1.44)
≥ Median % households with no car	1.02 (0.89, 1.16)	0.99 (0.74, 1.33)	0.94 (0.84, 1.04)	0.91 (0.73, 1.14)	1.09* (1.01, 1.19)	1.46*** (1.20, 1.78)
≥ Median % public assistance	0.96 (0.84, 1.10)	1.02 (0.75, 1.40)	0.89* (0.80, 0.99)	0.95 (0.76, 1.19)	1.04 (0.96, 1.13)	1.32** (1.08, 1.62)
Deprivation quartile 2 versus 1	1.04 (0.89, 1.21)	1.84 (0.98, 3.46)	0.98 (0.87, 1.11)	1.28 (0.82, 2.01)	1.05 (0.96, 1.15)	1.32 (0.89, 1.95)
Deprivation quartile 3 versus 1	0.91 (0.76, 1.08)	1.45 (0.81, 2.58)	0.90 (0.78, 1.04)	0.97 (0.65, 1.45)	1.01 (0.90, 1.13)	1.36 (0.96, 1.94)
Deprivation quartile 4 versus 1	1.27* (1.01, 1.59)	1.62 (0.93, 2.81)	0.98 (0.82, 1.18)	1.13 (0.77, 1.64)	1.34*** (1.19, 1.52)	1.71** (1.21, 2.40)
Deprivation quartile, *P* value	*P* = 0.055	*P* = 0.227	*P* = 0.512	*P* = 0.239	*P* < .001***	*P* < .001***

*<.05, **<.01, ***<.001.

**Table 3:** Unstratified analysis—associations of neighborhood SES characteristics with indicators of prostate cancer severity (GEE) adjusted for age, race, and diagnosis year.

Effect	Tumor aggressiveness		High stage		High Gleason	
	OR (CI)	*P* value	OR (CI)	*P*-value	OR (CI)	*P*-value
African American race/ethnicity	1.31 (1.16, 1.47)	<.001	1.27 (1.17, 1.39)	<.001	1.37 (1.27, 1.47)	<.001

≥ Median % neighborhood poverty	0.99 (0.87, 1.12)	0.853	0.93 (0.84, 1.02)	0.126	1.09 (1.01, 1.17)	0.028
African American race/ethnicity	1.32 (1.15, 1.50)	<.001	1.32 (1.20, 1.46)	<.001	1.31 (1.21, 1.42)	<.001

≥ Median % household income <$30,000	1.05 (0.93, 1.19)	0.446	1.00 (0.91, 1.10)	0.998	1.10 (1.02, 1.19)	0.014
African American race/ethnicity	1.28 (1.12, 1.46)	<.001	1.27 (1.15, 1.41)	<.001	1.31 (1.20, 1.42)	<.001

< Median % high school education	1.12 (1.00, 1.27)	0.054	1.01 (0.92, 1.11)	0.802	1.10 (1.02, 1.19)	0.010
African American race/ethnicity	1.24 (1.09, 1.41)	<.001	1.27 (1.15, 1.39)	<.001	1.31 (1.21, 1.42)	<.001

≥ Median % female head of household with dependent child(ren)	1.02 (0.90, 1.16)	0.727	0.94 (0.85, 1.04)	0.217	1.09 (1.01, 1.17)	0.030
African American race/ethnicity	1.29 (1.13, 1.48)	<.001	1.31 (1.19, 1.45)	<.001	1.31 (1.21, 1.42)	<.001

≥ Median % households with no car	1.01 (0.90, 1.14)	0.845	0.93 (0.85, 1.03)	0.161	1.13 (1.05, 1.22)	0.001
African American race/ethnicity	1.30 (1.14, 1.48)	<.001	1.32 (1.19, 1.45)	<.001	1.28 (1.18, 1.40)	<.001

≥ Median % public assistance	0.97 (0.85, 1.09)	0.576	0.89 (0.81, 0.99)	0.026	1.08 (1.00, 1.16)	0.063
African American race/ethnicity	1.33 (1.17, 1.52)	<.001	1.34 (1.22, 1.49)	<.001	1.32 (1.21, 1.43)	<.001

Deprivation quartile		0.064		0.245		<.001
Deprivation quartile 2 versus 1	1.07 (0.92, 1.24)	0.390	0.99 (0.88, 1.12)	0.882	1.06 (0.98, 1.16)	0.165
Deprivation quartile 3 versus 1	0.94 (0.80, 1.11)	0.470	0.89 (0.78, 1.02)	0.083	1.03 (0.93, 1.15)	0.543
Deprivation quartile 4 versus 1	1.19 (0.99, 1.43)	0.068	0.99 (0.86, 1.14)	0.927	1.36 (1.22, 1.51)	<.001
African American race/ethnicity	1.20 (1.03, 1.39)	0.020	1.27 (1.14, 1.42)	<.001	1.16 (1.06, 1.26)	<.001

*<.05, **<.01, ***<.001.
